# Degradation of norfloxacin by copper-doped Bi_2_WO_6_-induced sulfate radical-based visible light-Fenton reaction†

**DOI:** 10.1039/d0ra07378d

**Published:** 2020-10-15

**Authors:** Xin Zhong, Wen-Ting Wu, Hao-Nan Jie, Wang-Ye Tang, Dan-Yan Chen, Tao Ruan, He-Ping Bai

**Affiliations:** Department of Environmental Engineering and Science, Beijing Normal University Zhuhai China zhongxin@bnu.edu.cn; College of Education for the Future, Beijing Normal University at Zhuhai Zhuhai China

## Abstract

In this work, a series of Cu(ii)-doped Bi_2_WO_6_ nanomaterials with good photo-response properties were facile synthesized and used to obtain efficient peroxymonosulfate (PMS) activation activity for norfloxacin (NOF) removal under visible LED light irradiation. It was found that Cu–Bi_2_WO_6_ presents superior catalytic performance for NOF degradation in comparison with pristine Bi_2_WO_6_, attributed to the partial substitution of Bi^3+^ by Cu ions. Moreover, the effects of experimental conditions were carefully investigated, including PMS concentration, catalyst dosage and initial pH, and the experimental data fitted well with the pseudo-first-order model. Experimental results implied that there was a synergic effect of visible LED light energy and the sulfate radical (SR)-Fenton reaction. Additionally, the 5Cu–Bi_2_WO_6_ nanomaterial presented the best degradation efficiency of 89.27% and exhibited high NOF degradation in 5 cycles with limited Cu leaching. Furthermore, EPR and radical quenching experiments were performed to identify the reactive oxygen species presented in the SR-photo-Fenton reaction. Finally, the major degradation intermediates of NOF were detected, and a possible degradation pathway was given. Thus, a mechanism of the significant photocatalytic activity enhancement by copper doping of the Bi_2_WO_6_ catalyst was proposed.

## Abstract 2

In this work, a series of Cu(ii)-doped Bi_2_WO_6_ nanomaterials with good photo-response properties were facile synthesized and used to obtain efficient peroxymonosulfate (PMS) activation activity for norfloxacin (NOF) removal under visible LED light irradiation.

## Graphical abstract

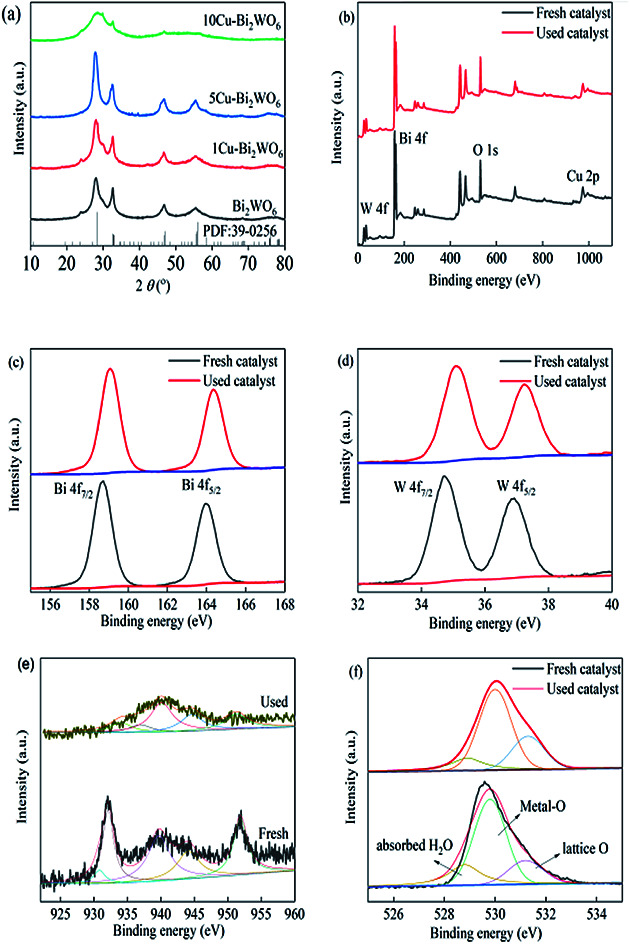

## Introduction

Norfloxacin, a fluoroquinolone antibiotic, is widely used for preventing serious diseases, which have been extensively detected in water such as hospital wastewater and urban wastewater.^1–3^ The residual concentration of NOF detected in water not only threatens the safety of human living environment and health, but also leads to drug resistance of bacteria and disturbs the living conditions of microorganisms. However, residual antibiotics are very difficult to remove by traditional water treatment methods, such as activated sludge, absorption and coagulation–sedimentation.^4^ Thus, various efforts have been made to find effective and practical applicable technology to limit the negative residual of NOF.^5–7^ Visible light-driven photocatalysis technology has attracted increasing attention owing to its effective potential in the removal of refractory organics and its environmental friendliness, exhibiting potential as a green technology due to the formation of highly reactive species under the stimulation of visible light.^8–10^ The photocatalysts play a great role in the degradation efficiency and practical applications for water treatment.

Recently, Bi-based materials have been reported to be effective photocatalysts or heterogeneous catalysts for PMS activation owing to their narrow band gap and unique hoist structure.^11–13^ Among these materials, Bi_2_WO_6_ is widely used and is considered to be a promising photocatalyst due to its good photo-response ability and excellent stability during wastewater treatment.^14^ However, the photocatalytic activity is always unsatisfactory when using pristine Bi_2_WO_6_ materials due to the rapid recombination of photo-generated carriers. Thus, further attempts have been investigated to develop new approaches to improve the photocatalytic efficiency of Bi_2_WO_6_, such as doping metal/non-metal ions in to the lattice structure^15–17^ and formation of heterojunctions with other semiconductors.^18–20^ Additionally, the doping strategy could effectively separate the photogenerated holes and electrons, inhibiting the recombination between the photogenerated carriers. Hence, the introduction of transition metal elements is considered to be an efficient method to improve the photocatalytic activity of Bi_2_WO_6_. For example, Bi_2_WO_6_ has been decorated with other metal ions, such as Ag,^21^ Zn,^22^ Mg,^23^ Sm,^24^ and Ti,^25^ to manipulate the excitation and separation of photogenerated carriers; this can improve its photocatalytic activity for redox applications. Doping of copper into Bi_2_WO_6_ has also been proved to exhibit excellent photocatalytic activity under visible light.^26^ Gao *et al.* reported that the photocatalytic activity of Cu-doped Bi_2_WO_6_ was 92% for the degradation of phenol.^27^ Tan *et al.* reported that modifying Bi_2_WO_6_ with Cu significantly increased the photocatalytic activity of the Bi_2_WO_6_ and afforded complete RhB removal efficiency.^28^ However, the light source was a high-power xenon lamp or gold halide lamp, and the reaction time was long.

Herein, peroxymonosulfate/persulfate was introduced in the photocatalysis process; it is considered to be an environmentally friendly oxidant, as reported in many previous studies.^29–31^ Due to the presence of PMS in the photocatalysis process, sulfate radicals and hydroxyl radicals would be generated through the heterogeneous SR-Fenton activation of PMS. On the other hand, the PMS would function as the electron acceptor, effectively preventing the recombination of photogenerated holes and electrons and improving the degradation efficiency compared to the photocatalysis process.^32^ It was expected that the synergistic effect of the photocatalysis process and the heterogeneous SR-Fenton reaction would occur in the SR-photo-Fenton process. Different kinds of photocatalysts that have been employed in the SR-photo-Fenton process are listed in Table S1.† To the best of our knowledge, there has been little study on the application of an LED lamp (460–470 nm) as the light source in a photocatalytic process using Cu-doped Bi_2_WO_6_ materials with the introduction of PMS.

Considering the synergism effect and good catalytic performance of the copper species, a Cu-doped Bi_2_WO_6_ nanomaterial was used for the activation of PMS under visible LED light. The Cu–Bi_2_WO_6_ nanomaterials were successfully synthesized through a simple one-pot-step hydrothermal method. The morphologies, crystal microstructures and chemical properties of the catalysts were systematically evaluated. Then, the NOF removal performance with Cu–Bi_2_WO_6_ as the photocatalyst coupled with PMS under visible LED light irradiation was studied in detailed experiments. Radical quenching experiments and electron paramagnetic resonance (EPR) experiments were conducted. The intermediates during the NOF degradation process were detected by LC/MS, and a degradation pathway is proposed. The results show that the Cu–Bi_2_WO_6_ nanomaterials showed better photocatalytic performance than pristine Bi_2_WO_6_ catalyst alone. Furthermore, on the basis of the obtained results, the possible reaction mechanism was also illustrated.

## Experimental

### Synthetic procedures

All the reagents were of analytical grade and were used without further purification. The Cu–Bi_2_WO_6_ nanomaterials were prepared by a facile one-pot-step hydrothermal method which was used in previous work.^33^ A schematic of the synthesis procedure is shown in Fig. S1.† 2 mmol [Bi(NO_3_)·5H_2_O + different contents of Cu(NO_3_)_2_·3H_2_O] and 1 mmol Na_2_WO_4_·2H_2_O were dissolved in 10 mL of ethylene glycol, respectively. The two solution was mixed under magnetic stirring; then, 30 mL ethanol and 0.3 g of glucose were added to the mixed solution. The solution was transferred into a 100 mL Teflon-lined stainless-steel autoclave and maintained at 160 °C for 12 h. The solid was centrifuged and washed with deionized water and ethanol, then dried at 70 °C for 24 h. Finally, different amounts of Cu loading were obtained with different molar ratios of Cu and Bi, denoted as Bi_2_WO_6_, 1Cu–Bi_2_WO_6_, 5Cu–Bi_2_WO_6_ and 10Cu–Bi_2_WO_6_.

### Photocatalytic activity experiments

In a typical experiment, an LED lamp (30 W, 460 nm, Xujia Company, China) was employed as the light source.^34^ 0.1 g Cu–Bi_2_WO_6_ photocatalyst was added to 50 mL of NOF solution (15 mg L^−1^). Before the light was turned on, the mixture was magnetically stirred in the dark for 60 min. Then, PMS was added to the system, and the LED light was turned on. The sample was taken out and filtered through 0.22 μm membrane filters, and the concentrations of NOF and TOC were measured by HPLC (Agilent 1100 LC/MSD, C18 column) and TOC (Elementar Vario) analyzers, respectively. Each sample was measured three times, and the average values are shown in the figures. Electron spin resonance (ESR, JES FA200, JEOL) was used to measure the intensity of the free radicals. The copper leaching measurements were performed using an ICP-MS (Agilent 7000). The intermediate products from the NOF degradation were identified using liquid chromatograph-mass spectrometry (LC-MS, Thermo Fisher, TSQ Endura). In the batch experiments, the degradation of NOF was fitted well using a pseudo-first-order kinetic model, which can be expressed as eqn (1).

1 ln *C*/*C*_0_ = −*k*_app_ × *t*

where *C* refers to the concentration of NOF at time *t*, *C*_0_ refers to the initial NOF concentration, *k*_app_ refers to the kinetic rate constant, and *t* refers to the reaction time.

### Characterization of the as-prepared samples

Power X-ray diffraction (XRD) patterns were obtained by a Bruker D8 ADVANCE with graphite monochromatic Cu Kα radiation (*λ* = 0.154 nm), where the accelerating voltage was fixed at 40 kV and the current was 30 mA. The particle sizes and morphologies of the as-synthesized catalysts were characterized through transmission electron microscopy (TEM, FEI Talos F200S) and scanning electron microscopy (SEM, Hitachi SU8220) equipped with an energy dispersive spectroscopy (EDS) instrument to determine the element distributions. X-ray photoelectron spectroscopy (XPS) was conducted to obtain the surface chemical information with a Thermo Fisher ESCALAB 250Xi. A UV-Vis spectrophotometer (Shimadzu UV-3600 Plus) was also employed. The BET surface areas and particle sizes of the samples were analysed by N_2_ adsorption–desorption technology on a TriStar II 3020.

## Results and discussion

### Characterization of the samples

**Fig. 1:**
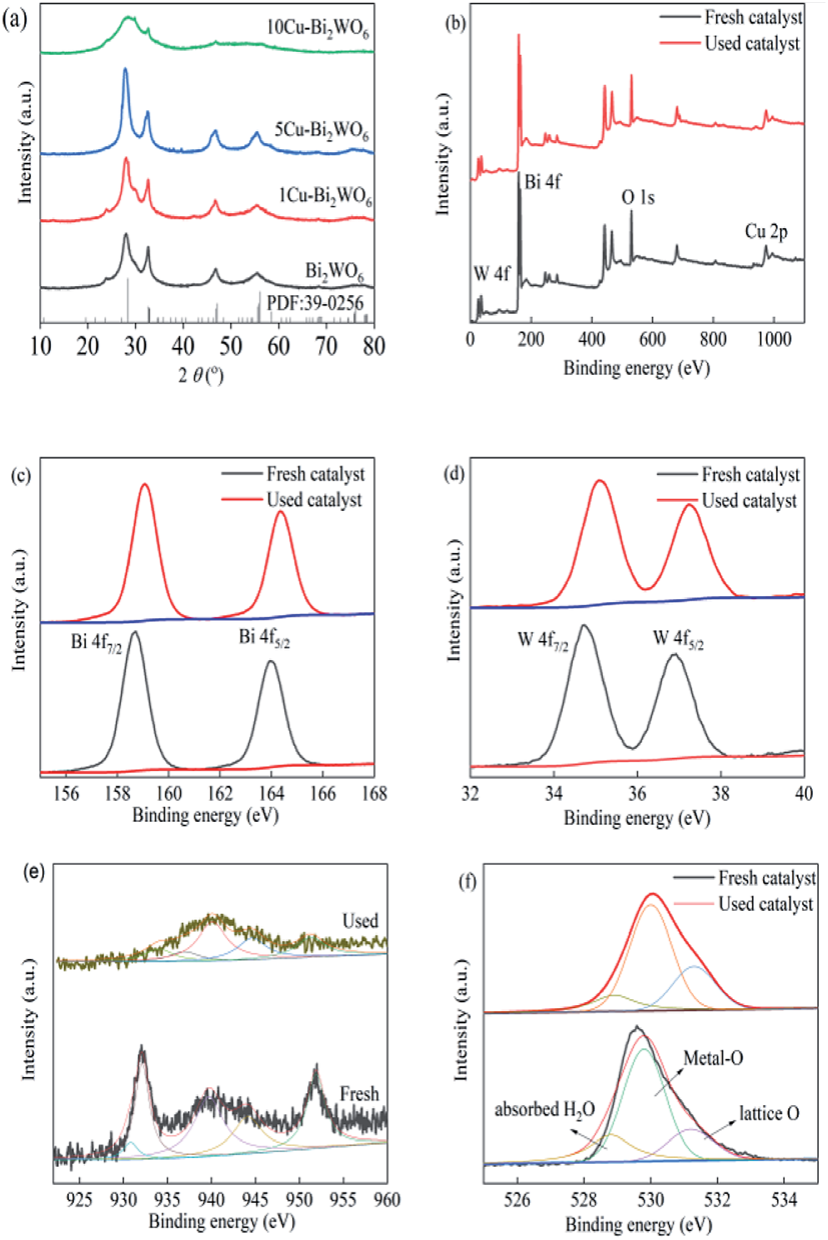
(a) XRD patterns of Bi_2_WO_6_ and the Cu–Bi_2_WO_6_ photocatalysts; XPS spectra of 5Cu–Bi_2_WO_6_ before and after the reaction: (b) survey, (c) Bi 4f, (d)W 4f, (e) Cu 2p and (f) O 1s.

The chemical crystallinities and purities of the synthesized Bi_2_WO_6_ and copper-doped Bi_2_WO_6_ materials were investigated by XRD, and the results are shown in Fig. 1. The identified diffraction peaks of 28.1°, 32.6°, 46.9°, 54.7° and 76.3° fitted well with the crystal structure of Bi_2_WO_6_ (JCPDS no. 39–0256, *a* = 0.5457 nm, *b* = 1.6435 nm, *c* = 0.5438 nm), corresponding to the indices of the (113), (200), (026), (313) and (402) planes, respectively.^35^ After copper was decorated on the surface of pristine Bi_2_WO_6_, the XRD pattern peaks showed no obvious changes in comparison with pristine Bi_2_WO_6_, evidencing that the host structure of Bi_2_WO_6_ remained stable and was not affected by the introduction of copper ions. The diffraction peaks were observed to slightly shift to left-side angles in the range of 0.13–0.25° with increased copper loading. This could be assigned to enlarged inter-planar spacing due to the partial substitution of Bi^3+^ ions by copper ions, whereas the ionic radius of copper ions (0.073 nm) was smaller than that of Bi ions (0.103 nm). It was observed that the copper doping strategy did not change the crystal structure of the host Bi_2_WO_6_ lattice.^36^ It was reasonably inferred that copper ions were squeezed into the lattice, resulting in the expansion of the crystalline lattice. However, a large amount of Cu ions would lead to decreased crystallinity and low intensity of the characteristic peaks. Meanwhile, it can be seen that no impurity diffraction peaks for copper oxides or other phases were obtained, which suggests that the doped copper element caused no significant changes in the Bi_2_WO_6_ structure owing to its low content and good dispersion in the structure of the host.

The XPS spectra of the 5Cu–Bi_2_WO_6_ catalyst before and after the reaction are shown in Fig. 1b–f. It was observed that the Cu–Bi_2_WO_6_ was composed of four elements, *i.e.* Bi, W, O and Cu. Fig. 1c shows that there were two peaks centered at 158.6 eV and 163.9 eV, which were attributed to Bi 4f_7/2_ and Bi 4f_5/2_, respectively. Herein, two peaks fixed at 36.9 eV and 34.7 eV were resolved into the contributions of W 4f_5/2_ and W 4f_7/2_, proving the existence and presence of W^6+^. The high-resolution spectrum of O 1s could be separated into three peaks at 528.8 eV, 529.9 eV and 531.2 eV, which can be attributed to the oxygen species of lattice oxygen, Bi–O or Cu–O bonds, and adsorbed hydroxyl groups or H_2_O, respectively. After the SR-photo-Fenton reaction, the area proportion of oxygen species to the adsorbed hydroxyl groups was higher than in the fresh catalyst, demonstrating the participation of O species on the surface with the formation of photogenerated holes and electrons.^37^ The XPS spectrum of Cu 2p is shown in Fig. 1d, and it can be separated into five peaks. The peak centered at 951.8 eV could be considered as due to the presence of Cu 2p_1/2_, and the peaks at 943.9 eV and 939.7 eV would be recognized as the shake-up satellite peaks of copper ions; the peaks appearing at 930.8 eV and 932.1 eV can be assigned to Cu 2p_3/2_. The XPS results in the used catalyst were similar to those in the fresh catalyst. However, the peak proportion of reduced copper species was much sharper than that of the fresh catalyst, evidencing the redox cycling of Cu(i)/Cu(ii), which participated in the reaction. With the introduction of copper species, surface oxygen vacancies were also produced due to the substitution of Bi ions, leading to extrinsic oxygen deficiency and enhanced oxide ion conductivity.

**Fig. 2:**
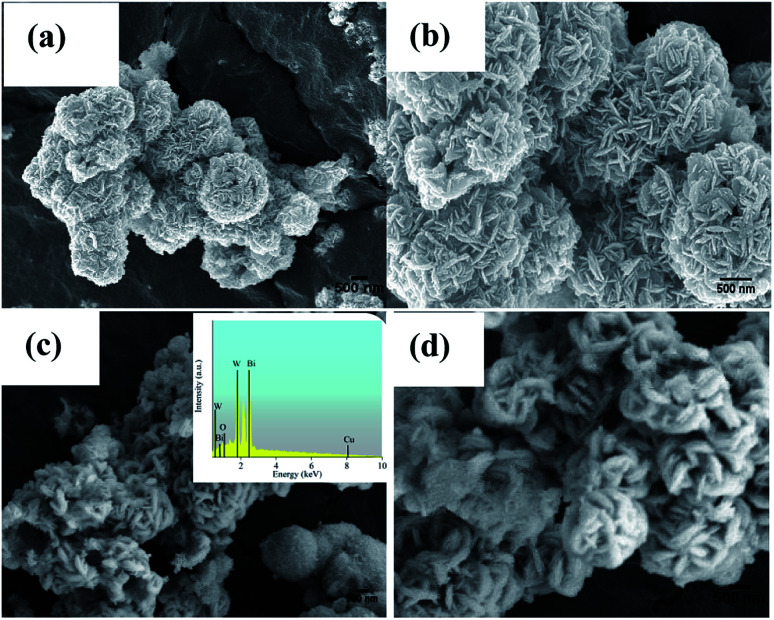
SEM images of the (a and b) Bi_2_WO_6_ and (c and d) 5Cu–Bi_2_WO_6_ samples.

SEM was employed to obtain the surface morphologies and microstructures of the Bi_2_WO_6_ and 5Cu–Bi_2_WO_6_ materials, and the results are shown in Fig. 2. The morphology of the pristine Bi_2_WO_6_ catalysts exhibited hierarchical flower-like microstructures which were composed of nanosheets and nano-sticks. The average diameter of the single flower-like structures was approximately 1.5–2 μm. On the other hand, after the Cu ion doping, the morphologies of the synthesized samples changed greatly. It was observed that the Cu ions presented monoclinic phase and that the morphology changed to a wool-ball-like morphology. After the Cu ions were doped, many small particles were absorbed on the surface of the primary nanostructures and the morphology became irregular. The catalyst surface was much rougher compared with that of pristine Bi_2_WO_6_ because of the Cu particles deposited on the surface of the Bi_2_WO_6_ nanoflowers with damaged fragments of nano-sticks. These results demonstrate that the partial replacement of Bi ions by copper ions did not change the host crystal structure; however, partial agglomeration occurred on the surface. Additionally, the multi-layered structure was observed to be thicker after doping of Cu into the host structure. In order to verify the chemical composition of the catalyst, EDS spectra were used; the results revealed that the Cu–Bi_2_WO_6_ material was composed of Bi, W, Cu and O elements, indicating that the copper ions were successfully decorated on the surface of the catalyst.

**Fig. 3:**
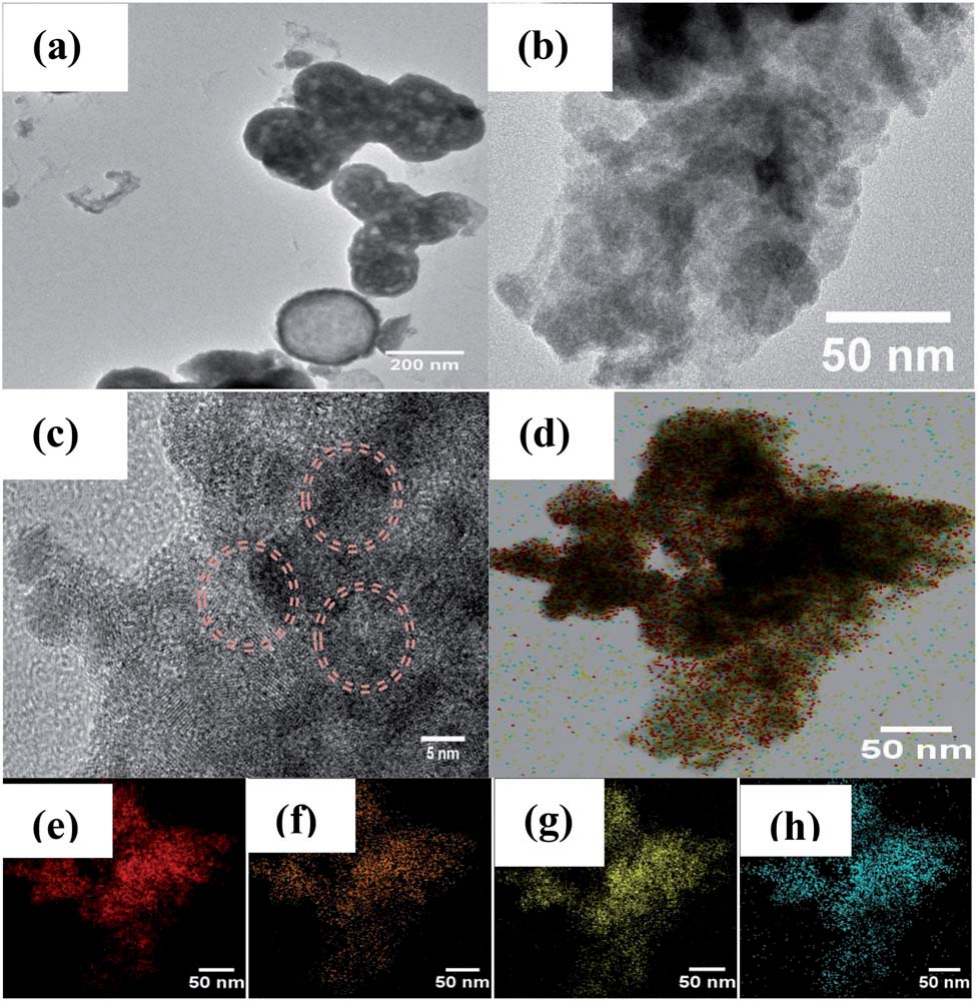
TEM images of (a) Bi_2_WO_6_, (b and c) 5Cu–Bi_2_WO_6_; (d–h) mapping images of 5Cu–Bi_2_WO_6_, (e) Bi, (f) W, (g) O, and (h) Cu.

The TEM images in Fig. 3a show that the pristine Bi_2_WO_6_ materials present both microsphere and rectangle morphologies with stick-like nanocomposites in the size diameter range of 260–270 nm. As shown in Fig. 3b, it can be clearly observed that porous structures of Bi_2_WO_6_ nanosheets were obtained and that the Cu particles were present on the surface of the Bi_2_WO_6_; the lattice spacings of 0.274 nm and 0.268 nm can be attributed to the (200) and (002) planes of Bi_2_WO_6_.^38^

The results further evidence that the doped copper ions were finely incorporated in the host Bi_2_WO_6_ lattice. In order to describe the elemental distribution of copper, an elemental mapping analysis was performed, which proved the existence of Bi, W, O and Cu elements. Moreover, the mapping results showed the homogeneous distribution of copper species on the surface of the Bi_2_WO_6_ materials, which were in close contact to the surface.

**Fig. 4:**
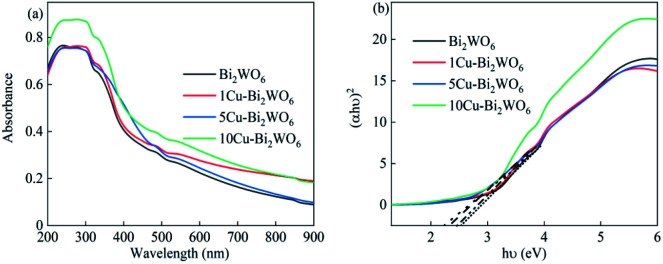
(a) UV-Vis absorption spectra and (b) corresponding Tauc plots of (*αhυ*)^2^*versus* (*hυ*) of pristine Bi_2_WO_6_ and the Cu–Bi_2_WO_6_ catalysts.

The optical properties of Cu–Bi_2_WO_6_ were explored using UV-Vis spectra, which are shown in Fig. 4. For the pristine Bi_2_WO_6_ material, an absorption edge was observed around 420 nm. After the copper doping into the structure of Cu–Bi_2_WO_6_, the absorption edge was significantly enhanced and red shifted to the visible light region; this can be attributed to the charge transfer between the copper species and host matrix.^39^ For a crystalline semiconductor, the band gap was calculated based on the absorption spectra according to the equation *ahv* = *A*(*hv* − *E*_g_)*n*, where *a*, *hv*, *E*_g_, and *A* refer to the absorption coefficient, the light frequency, the band gap, and a constant, respectively.^40^ The band gaps of the Cu-doped Bi_2_WO_6_ with different contents of copper ions could be estimated to be 2.61 eV, 2.57 eV, 2.43 eV, and 2.37 eV, respectively. In contrast to the pristine Bi_2_WO_6_ material, all the Cu-doped Bi_2_WO_6_ materials showed that with increasing copper loading, the band gap slightly decreased. The presence of copper ions in Bi_2_WO_6_ did not affect the position of the valence band edge; however, it introduced new energy levels of the copper ions into the band gap of Bi_2_WO_6_, leading to a decreased band gap. These results can be attributed to the charge-transfer transitions between the copper ions and the Bi_2_WO_6_ host structure.

**Fig. 5:**
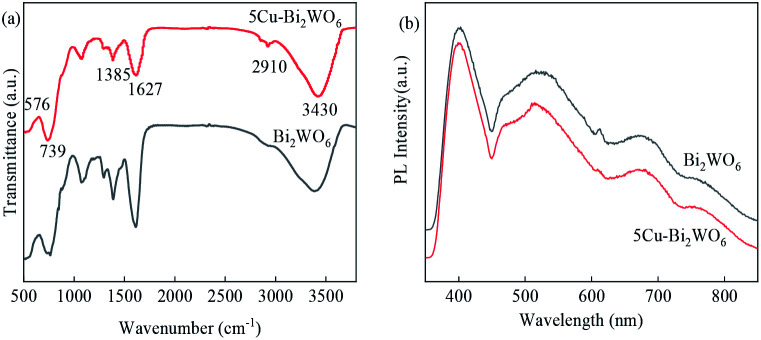
(a) FTIR spectra of Bi_2_WO_6_ and 5Cu–Bi_2_WO_6_, (b) PL spectra of Bi_2_WO_6_ and the 5Cu–Bi_2_WO_6_ catalyst.

The FTIR spectra of the synthesized Bi_2_WO_6_ and 5Cu–Bi_2_WO_6_ catalysts are shown in Fig. 5a. The absorption peaks observed at around 3430 cm^−1^ and 1627 cm^−1^ can be attributed to stretching vibrations of hydroxyl groups, evidencing the existence of absorbed water. The observed peaks at 1454 cm^−1^ can be attributed to the N–O bond bending. The presence of Bi–O, W–O and W–O–W was confirmed from the broad absorption peaks in the range of 500–1400 cm^−1^, with 576 cm^−1^ for Bi–O stretching, 739 cm^−1^ for W–O stretching and 1385 cm^−1^ for W–O–W stretching vibrations. The FTIR spectra of the prepared Cu–Bi_2_WO_6_ nanomaterials matched well with that of the pristine Bi_2_WO_6_ nanoflowers. In order to investigate the transfer and recombination of photogenerated carriers, the PL spectrum was introduced and is shown in Fig. 5b. It can be observed that the emission peak intensity of pristine Bi_2_WO_6_ was much higher than that of the Cu–Bi_2_WO_6_ materials, indicating that the recombination of photogenerated carriers was quicker. The results suggest that recombination of the photogenerated carriers was prevented due to the doping of copper into the host structure of Bi_2_WO_6_, resulting in better degradation efficiency.

The BET surface areas and particle sizes of Bi_2_WO_6_ and the Cu–Bi_2_WO_6_ materials were investigated by N_2_ adsorption–desorption analysis. The N_2_ adsorption–desorption isotherm and the pore size distribution are shown in Fig. S2.† It was observed that the hysteresis loops of the isotherms for Bi_2_WO_6_ were type IV with an H3 loop, demonstrating the existence of mesoporous structures.^41^ Moreover, the BET surface area of the Bi_2_WO_6_ structure was affected by copper doping. With the copper doping, the surface area slightly increased from 46.8 m^2^ g^−1^ to 52.2 m^2^ g^−1^; the average pore size was within the range of 12–13 nm, which would facilitate the mass transfer of large chemical molecules. Surface defects in the Bi_2_WO_6_ structure were created by the copper doping strategy. The BET surface area increased with increasing copper element doping, which provided more active sites and facilitated the photo-response ability in the further photocatalytic experiments.

### Photocatalytic performance

**Fig. 6:**
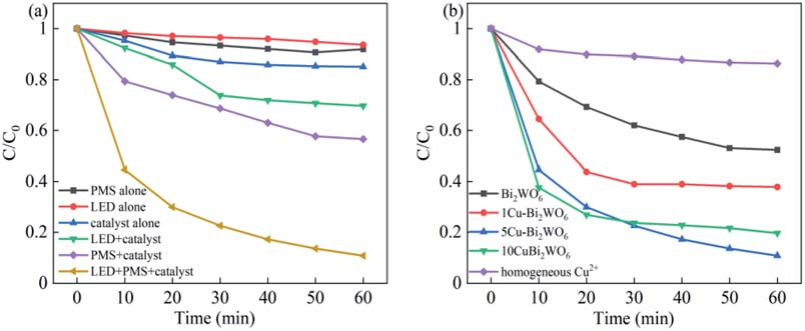
(a) Different reaction systems of NOF over the 5Cu–Bi_2_WO_6_ photocatalysts and (b) catalysts with different Cu loadings in the photocatalytic degradation.

To demonstrate the photocatalytic activities of the Cu–Bi_2_WO_6_ photocatalyst, the photocatalytic degradation of NOF under different experimental conditions in the SR-photo-Fenton reaction was investigated, and the results are shown in Fig. 6. The NOF removal efficiency was almost negligible when using PMS alone owing to the limited self-decomposition of PMS. About 14.88% and 6.36% NOF removal were detected by the catalyst adsorption experiment and when using visible LED light irradiation alone, respectively. When Cu–Bi_2_WO_6_ and PMS were both presented in the NOF solution under dark conditions, *i.e.* the SR-Fenton process, the NOF removal efficiency was 43.42%, which indicates that Cu–Bi_2_WO_6_ can be an activator for PMS, leading to the production of free sulfate radicals. It was noted that 89.27% NOF removal efficiency was obtained in the SR-photo-Fenton reaction, which was nearly a 46% enhancement of NOF degradation compared with under dark conditions. Moreover, when PMS was added to the system, the rate constant *k*_app_ of NOF in the photocatalysis system (0.0344 h^−1^) was higher than the sum of that in the photocatalysis process (0.0064 h^−1^) and SR-Fenton systems (0.009 h^−1^). These results revealed that there is a synergistic effect between the two systems, proving the promotion effect of the electron acceptor on the photocatalytic process.

In order to evaluate the impact of the Cu content in the system, the effects of different Cu loadings were also explored and are shown in Fig. 6b. It was found that all Cu–Bi_2_WO_6_ samples exhibited higher NOF degradation efficiency than pristine Bi_2_WO_6_ (38.78%) in the photocatalysis reaction, where 5Cu–Bi_2_WO_6_ showed the best degradation efficiency (89.27%). When the copper loading was increased in the catalyst, the NOF degradation efficiency was enhanced, revealing the significant impact of Cu-doping on the catalytic activity. Furthermore, the rate constant of 5Cu–Bi_2_WO_6_ was about 3.28 times higher than that of pristine Bi_2_WO_6_ (0.0105 h^−1^), implying the good catalytic activity of Cu–Bi_2_WO_6_.

**Fig. 7:**
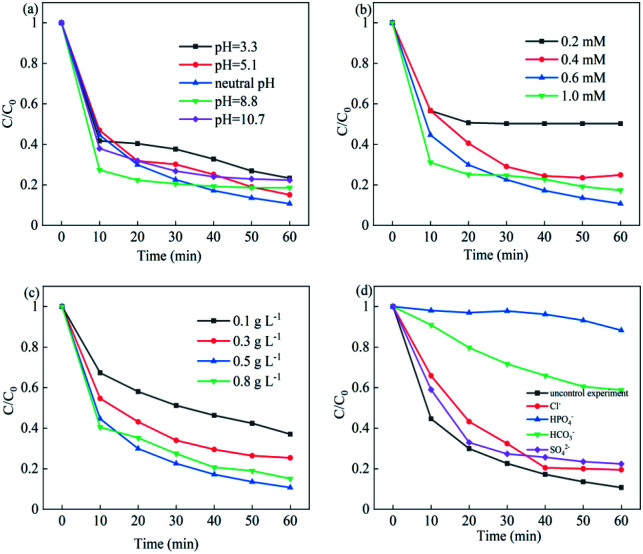
(a) Effects of pH; (b) PMS concentration; (c) catalyst dosage; and (d) inorganic anions on NOF degradation.

The effects of the parameters of initial pH, PMS concentration, catalyst dosage and various inorganic anions on the NOF degradation were systematically investigated. Fig. 7a showed the effects of initial pH on NOF removal; it can be observed that NOF could be efficiently degraded within 60 min in the pH range of 3.3–10.7. The rate constant increased from 0.0195 h^−1^ to 0.0344 h^−1^ under acidic pH from 3.3 to neutral pH; however, it slightly decreased when the pH was adjusted to 8.8 and further declined at a pH of 10.7. This can be attributed to the poor interaction with PMS oxidant due to the negatively charged surface of Cu–Bi_2_WO_6_ under basic conditions. In addition, the sulfate radicals would react with H_2_O or OH^−^, leading to the formation of hydroxyl radicals.^42^ On the other hand, the PMS oxidant could be decomposed to SO_5_^2−^ ions, which showed no catalytic activity.^43^ Therefore, the high efficiency of the Cu–Bi_2_WO_6_ systems is a great advantage in photocatalysis technology, and these systems would be efficient in a wide pH range.

2 SO_4_˙^−^ + H_2_O → ˙OH + SO_4_^2−^

3 SO_4_˙^−^ + OH^−^ → SO_4_^2−^ + ˙OH

4 HSO_5_^−^ → SO_5_^2−^ + H^+^

The effects of PMS concentration and Cu–Bi_2_WO_6_ dosage on the degradation of NOF are depicted in Fig. 7b and c, respectively. The degradation efficiency and rate constant of NOF increased with increasing PMS concentration; the best degradation efficiency was obtained at 0.6 mM PMS and then slightly decreased when the PMS concentration further increased to 1.0 mM. Indeed, the rate constant increased from 0.0083 h^−1^ to 0.0344 h^−1^ during the reaction time as the concentration of PMS increased from 0.2 mM to 0.6 mM, and the reaction rate decreased to 0.0225 h^−1^ at 1.0 mM PMS. These results could be due to the fact that more sulfate radicals would be generated when more PMS was added.^44^ PMS not only acted as the electron acceptor but was also employed as an oxidant in the reaction. However, it suffered from side reactions due to the annihilation of free radicals when the PMS concentration was too high. Finally, a PMS concentration of 0.6 mM was chosen for the following experiments.

As the catalyst dosage was added to the system, the degradation efficiency and rate constant of NOF increased correspondingly; however, they were inhibited by an overdose of the catalyst. The increment of the catalyst dosage from 0.1 to 0.5 g L^−1^ promoted the degradation efficiency of NOF from 62.93% to 89.27% in 60 min. The rate constant *k*_app_ varied from 0.014 h^−1^ to 0.0344 h^−1^; this can be attributed to the increased number of reactive sites, which leads to the generation of more free radicals. However, negligible improvement was gained when the catalyst dosage was over 0.5 g L^−1^; the rate constant decreased from 0.0344 h^−1^ to 0.0276 h^−1^. This was explained by the fact that the addition of more catalyst to the solution can result in more active sites for PMS activation. However, the degradation efficiency decreased when the catalyst dosage was over the optimized dosage; this would resist light absorption, leading to light scattering and diminishing of photons and further resulting in quenching reactions.^45^ Due to the economic concerns, the catalyst dosage of 0.5 g L^−1^ was chosen for the following experiments.

Additionally, the effects of common inorganic anions on NOF degradation were also explored, such as HPO_4_^−^, Cl^−^, SO_4_^2−^ and HCO_3_^−^. It was observed that the degradation of NOF was inhibited in the presence of HPO_4_^−^ (11.72%) and HCO_3_^−^ (41.17%) due to the intense scavenging effects of the two anions on the reactive oxidant species. For Cl^−^ (80.49%) and SO_4_^2−^ (77.6%) anions, the NOF degradation was slightly suppressed due to the quenching effect. The redox oxidization capacities of ˙HPO_4_, ˙Cl and CO_3_˙^−^ were much lower than those of sulfate radical and hydroxyl radicals, which functioned as quenching chemicals in the reaction, causing decreases in the NOF depredation efficiency and rate constant. The added inorganic anions had negative effects on the NOF removal owing to the free radical scavenging effect.^46–48^ Therefore, the free radicals would be quickly consumed with the addition of inorganic anions, leading to negative inhibition in the photocatalysis reaction.

5 HPO_4_^−^ + SO_4_˙^−^ → SO_4_^2−^ + ˙HPO_4_

6 Cl^−^ + SO_4_˙^−^ → SO_4_^2−^ + ˙Cl

7 Cl^−^ + ˙OH → ˙ClOH^−^

8 HCO_3_^−^ + SO_4_˙^−^ → SO_4_^2−^ + CO_3_˙^−^ + H^+^

9 HCO_3_^−^ + ˙OH → H_2_O + CO_3_˙^−^

**Fig. 8:**
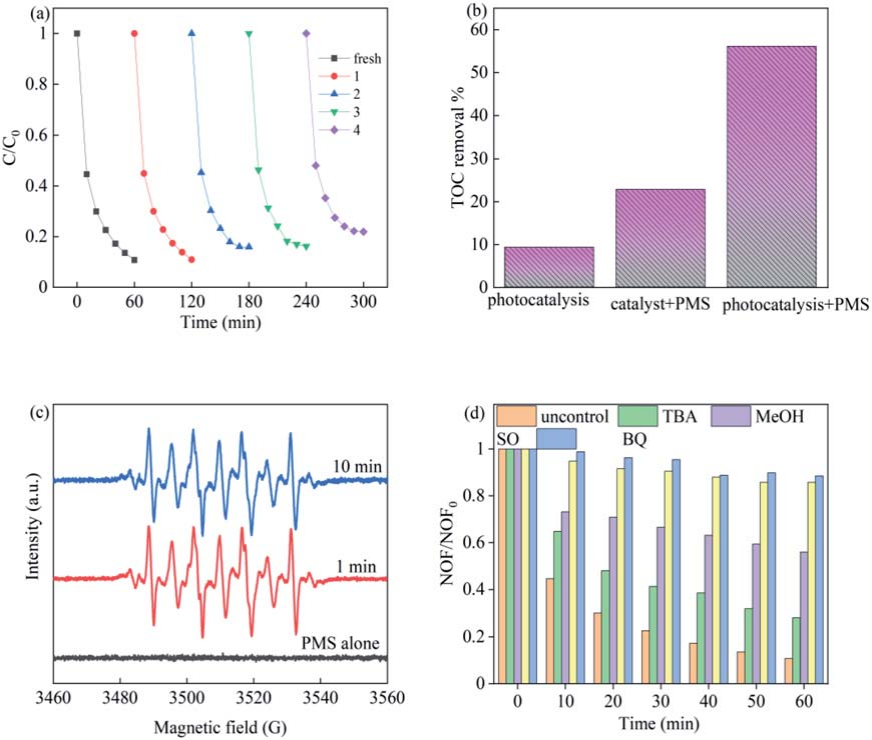
(a) Recycling experiments of Cu–Bi_2_WO_6_ in NOF degradation; (b) TOC experiment in different systems; (c) EPR experiment trapped by DMPO and (d) scavenging experiments.

To investigate the stability of Cu–Bi_2_WO_6_, XRD characterization of the used catalyst (Fig. S3†) illustrated that there were no significant changes in the location or the shape of characteristic diffraction peaks after the reactions. It was observed that the copper ion leaching was lower than 0.048 mg L^−1^ at neutral pH, which facilitated the cycling experiment, leading to better reusability of Cu–Bi_2_WO_6_. In order to illuminate whether the heterogeneous catalytic performance dominated the reaction, the effect of homogeneous Cu^2+^ (max leaching 0.048 mg L^−1^) was also investigated to activate PMS, as shown in Fig. 6b. The removal efficiency of NOF was observed to be no higher than 13.62%, validating that the catalyst played a great role in the reaction. The reaction was a heterogeneous catalytic reaction mechanism on the surface of Cu–Bi_2_WO_6_ to remove NOF from the photocatalysis system. As depicted in Fig. 8a, the degradation efficiency of NOF remained high in five successive runs in the 60 min reaction, revealing the superior cycling stability of Cu–Bi_2_WO_6_ materials. Moreover, a TOC experiment was conducted to evaluate the mineralization of NOF under optimal reaction conditions in 60 min. The TOC mineralization of NOF was 9.3%, 22.8% and 56.1% in the photocatalysis reaction, SR-Fenton reaction and SR-photo-Fenton reaction, respectively. The results showed that the mineralization ability was enhanced due to the synergism effect, which is in good agreement with the degradation efficiency of NOF.

### Radical quenching experiments and the possible pathways

To identify the possible free radicals produced in the photocatalysis system, an EPR experiment was conducted to verify the reactive oxidant species by using DMPO as spin trappers, as shown in Fig. 8c. Apparently, no EPR signals were detected by using PMS alone, evidencing that there was nearly no production of free radicals. Meanwhile in the SR-Fenton reaction under visible LED light irradiation, strong EPR signals were observed which can be recognized as the peaks of DMPO-X complexes (X for SO_4_˙^−^ and ˙OH), proving the formation of these free radicals.^49^ The peaks retained strong intensity for 10 min, which demonstrated the persistent generation of free radicals in the SR-photo-Fenton system.

In addition, quenching experiments were utilized to evaluate the contribution of these reactive oxygen radicals, such as TBA (*tert*-butyl alcohol) for ˙OH, MeOH (methanol) for SO_4_˙^−^ and ˙OH, BQ (*p*-benzoquinone) for ˙O_2_^−^ and SO (sodium oxalate) for holes. It can be seen in Fig. 8d that the degradation of NOF was significantly suppressed from 89.27% to 11.46% (BQ) and 14.28% (SO), respectively. The addition of TBA to the systems was found to cause slight suppression (72.07%), while the degradation efficiency reached 44.02% with the addition of MeOH. These results show that superoxide, holes and sulfate radicals are the major radicals in the reaction, whereas the hydroxyl radicals hardly affected the degradation efficiency or rate constant during the process.

**Fig. 9:**
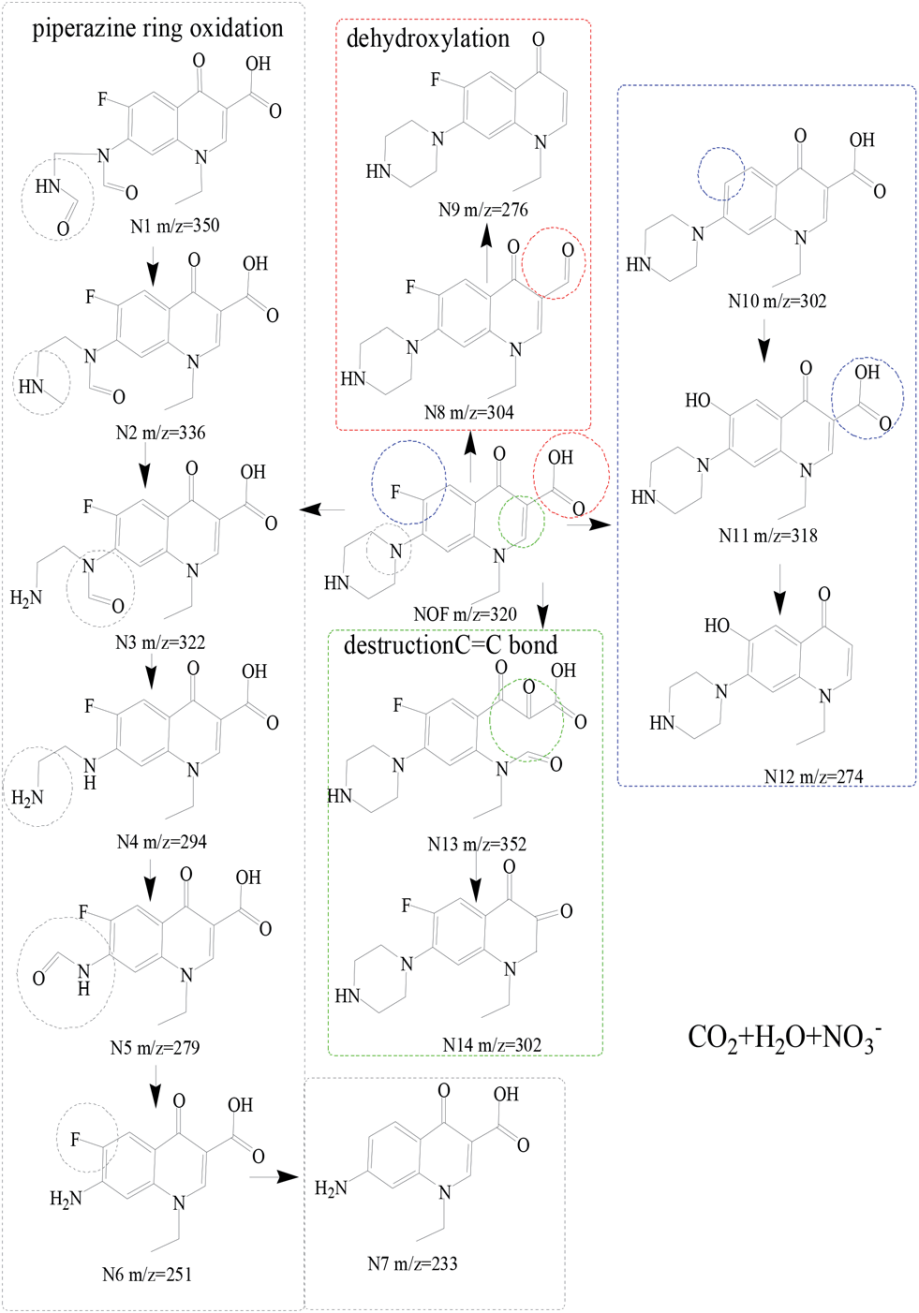
Photocatalytic degradation pathway of NOF in the photocatalysis system.

To identify the intermediates by NOF degradation, LC/MS technology was applied to obtain the degradation pathway for NOF in the photocatalysis reaction. About fourteen dominant intermediates were identified and are listed in Table S2,† and the possible degradation pathways are shown in Fig. 9. It was assumed that the NOF went through four possible pathways. At first, NOF was composed of the intermediates of N1 (*m*/*z* = 350) due to the destruction of the piperazinyl group by oxidation.^50^ Then, with the release of O atom, N2 (*m*/*z* = 336) was formed due to the free radical attacks, which further generated N3 (*m*/*z* = 322). Simultaneously, N4 (*m*/*z* = 294) and N5 (*m*/*z* = 279) were produced by the elimination of –C=O bonds and –NH_2_ groups, which gradually changed to N6 (*m*/*z* = 251) and N7 (*m*/*z* = 233). On the other hand, N8 (*m*/*z* = 304) was generated through a dehydroxylation process by direct photocatalytic degradation, which gradually produced N9 (*m*/*z* = 276) *via* the cleavage of –COOH bonds.^51^ Simultaneously, a defluorination process could occur in the reaction, leading to the formation of N10 (*m*/*z* = 302). Then, N10 went through a hydroxylation and decarboxylation process with the production of N11 (*m*/*z* = 318) and N12 (*m*/*z* = 278).^52^ Finally, the cleavage of C=C bonds would generate N13 (*m*/*z* = 352) owing to the attack of free radicals on the C=C bonds, which further changed to N14 (*m*/*z* = 302) with the departure of carboxyl groups. Overall, the degradation pathway of NOF can be concluded to be based on the processes of piperazinyl dealkylation, hydroxylation and oxidation of hydroxyl groups.^53^

### Photocatalytic degradation mechanism of NOF

Under visible LED light irradiation, the Cu–Bi_2_WO_6_ samples can absorb visible LED light; this excites electrons to transfer from the VB to the CB, leaving holes in the VB and promoting electron migration to the CB. The transfer of photogenerated carriers in Cu–Bi_2_WO_6_ facilitated the separation of the carriers and prevented the recombination of holes and electrons. To explore the mechanism of the enhanced photocatalysis activity, the relative band positions of Cu–Bi_2_WO_6_ were investigated. The band values of the valence band (VB) and conduction band (CB) of the synthesized semiconductors can be calculated from the equations:

10 *E*_VB_ = *χ* − *E*_e_ + 0.5*E*_g_

11 *E*_VB_ = *E*_CB_ + *E*_g_

where *χ* is the electronegativity of the semiconductor calculated from the geometric mean of the absolute electronegativity of the component atoms (6.39 eV for Bi_2_WO_6_), and *E*_e_ is the energy of free electrons based on the hydrogen scale (4.5 eV). The calculated *E*_VB_ values are 3.195 eV, while the calculated *E*_CB_ values are 0.585 eV. As the CB value of pure Bi_2_WO_6_ is higher than the redox potential of O_2_/˙O_2_^−^ (−0.33 eV *vs.* NHE), the superoxide radicals could not be directly generated by the photoexcited electrons. However, in the quenching experiments, it was clearly shown that superoxide radicals were present and played a great role in the photocatalysis process. It was reasonable to expect that the doping of Cu ions into the Bi_2_WO_6_ structure would facilitate the generation of superoxide radicals. As the Cu–Bi_2_WO_6_ photocatalyst was stimulated by visible LED light, the photoexcited electrons in the VB band transferred to Cu^2+^, leading to improved oxidative decomposition activity. Thus, the doped Cu ions would participate in the transfer of the photoexcited electrons to oxygen and the generation of superoxide radicals. On the other hand, the deposition of Cu on the surface of the photocatalyst of Bi_2_WO_6_ enhanced the separation of photogenerated carriers and promoted the photodegradation performance.

**Fig. 10:**
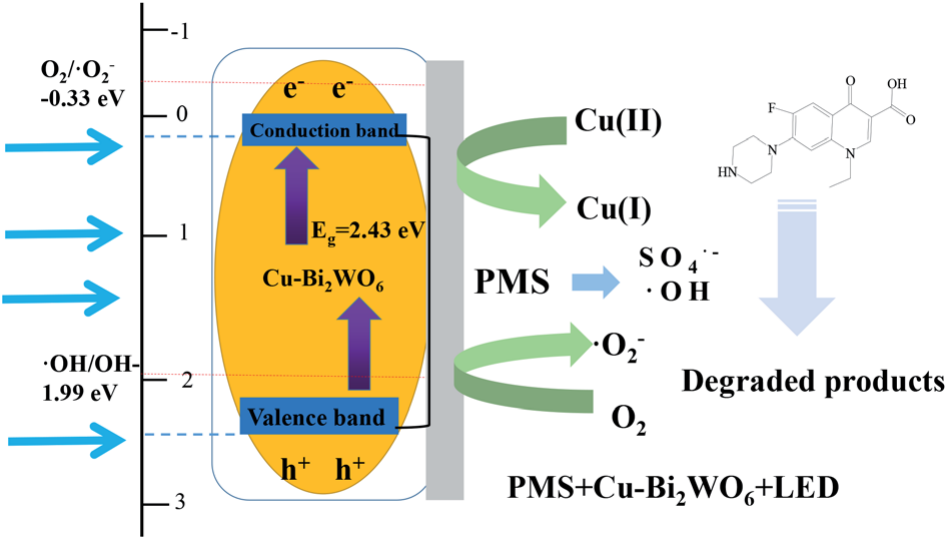
Mechanism of photocatalysis over Cu–Bi_2_WO_6_.

Based on the above analysis and experimental results, the reaction mechanism of NOF degradation in the SR-photo-Fenton systems was proposed and is shown in Fig. 10. With the introduction of PMS, the sulfate radicals and hydroxyl radicals were generated through the activation of the heterogeneous catalyst and stimulation of visible LED light.^54^ The copper doping in the structure of Bi_2_WO_6_ also facilitated the decomposition of PMS, further enhancing the formation of free radicals, accompanied with the redox transition of the Cu(ii)/Cu(i) pairs.

12 Cu(ii)–Bi_2_WO_6_ + HSO_5_^−^ → SO_4_˙^−^ + Cu(i)–Bi_2_WO_6_

13 Cu(i)–Bi_2_WO_6_ + HSO_5_^−^ → SO_5_˙^−^ + Cu(ii)–Bi_2_WO_6_

14 Cu–Bi_2_WO_6_ + visible light → Cu–Bi_2_WO_6_ + e^−^ + h^+^

Additionally, Bi_2_WO_6_ possesses a narrow band gap, which is responsible for the visible LED light reaction. Under LED light irradiation, electrons can be excited and transferred to the CB band, leading to the formation of photo-generated holes in the VB band. The generated holes and electrons would participate in the reaction with PMS and H_2_O due to the formation of sulfate radicals and hydroxyl radicals, which also promoted the regeneration of Cu(i)–Bi_2_WO_6_.

15 HSO_5_^−^ + e^−^ → SO_4_˙^−^ + OH^−^

16 H_2_O + h^+^ → ˙OH + H^+^

17 Cu(ii)–Bi_2_WO_6_ + e^−^ → Cu(i)–Bi_2_WO_6_

18 O_2_ + e^−^ → ˙O_2_^−^

The sulfate radicals could also react with OH^−^ and H_2_O, leading to the production of hydroxyl radicals. The results suggested that superoxide radicals and holes are major contributors to the decomposition of NOF. It was reasonably expected that the Cu doping strategy would efficiently promote the formation of superoxide through the reaction of photo-generated electrons. Finally, the reactive oxidizing species degraded NOF to produce CO_2_, H_2_O and NO_3_^−^. In order to verify the universal degradation ability of Cu–Bi_2_WO_6_, other organic chemicals were chosen as target pollutants, such as rhodamine B (RhB), orange II, methyl red (MR), and ciprofloxacin (CIP). The results showed that all the mentioned target pollutants were effectively degraded in the system (Fig. S5†). The degradation of RhB, orange II, MR and CIP was 100%, 100%, 96.43% and 66.51%, respectively. This SR-photo-Fenton system is suitable for the selective removal of organic pollutants and is expected to have good prospects in wastewater treatment.

## Conclusions

To summarize, copper-doped Bi_2_WO_6_ significantly enhanced NOF degradation in the SR-photo-Fenton reaction. It was observed to be composed of flower-like and wool-ball-like structures with good crystallinity and was expected to demonstrate good photocatalytic activity. A synergistic effect was observed between the SR-Fenton and photocatalysis systems. The optimal reaction was [PMS] = 0.6 mM and [catalyst] = 0.5 g L^−1^, and the catalyst can be applied in a wide pH range. Sulfate radicals, holes and superoxide were identified as major free radicals which contributed to the NOF removal. The TOC removal efficiency of NOF is about 56.1% over 5Cu–Bi_2_WO_6_ in 60 min. Meanwhile, 5Cu–Bi_2_WO_6_ also showed enhanced photocatalytic activity for different kinds of pollutants. In addition, the 5Cu–Bi_2_WO_6_ photocatalyst shows excellent photocatalytic performance and great stability for the degradation of diverse organic pollutants in water. Finally, a NOF degradation pathway was given and a synergism mechanism was proposed in the SR-photo-Fenton reaction.

## Conflicts of interest

There are no conflicts to declare.

## Supplementary Material

RA-010-D0RA07378D-s001
