# Supplementary material for: Degradation of norfloxacin by copper-doped Bi2WO6-induced sulfate radical-based visible light-Fenton reaction
Source: RSC Adv. 2020 Oct 15;10(62):38024–32. doi: 10.1039/d0ra07378d (PMC9057272; doi:10.1039/d0ra07378d)
Supplement: RA-010-D0RA07378D-s001 [file RA-010-D0RA07378D-s001.pdf]

Electronic Supplementary Material (ESI) for RSC Advances.
This journal is © The Royal Society of Chemistry 2020

Supplementary Material

Degradation of norfloxacin by sulfate radical-based visible light-Fenton by copper-doped Bi2WO6

Xin Zhong[1,2,*], Wen-Ting Wu[1], Hao-Nan Jie[1], Wang-Ye Tang[1], Dan-Yan Chen[1], Tao Ruan[1], He-ping Bai[1,2]

1.Department of Environmental Engineering and Science, Beijing Normal University, Zhuhai, Zhuhai, China;

2.College of Education for the Future, Beijing Normal University at Zhuhai, Zhuhai, China.

*Correspondence: zhongxin@bnu.edu.cn; Tel.: (+86-(0)756-3683012) (X. Zhong)

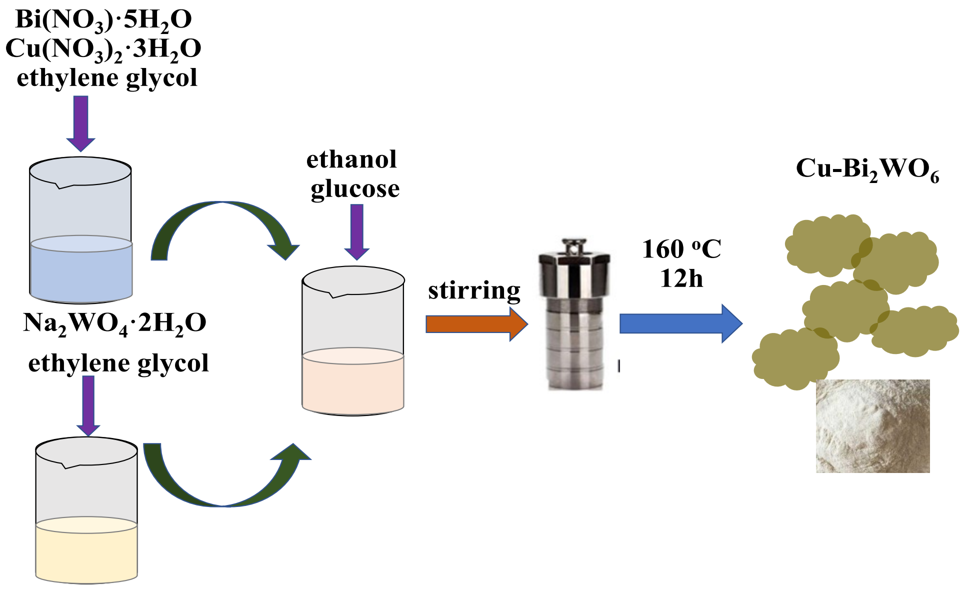

Figure S1 Schematic diagram of Cu-Bi_2WO_6 synthesis process.

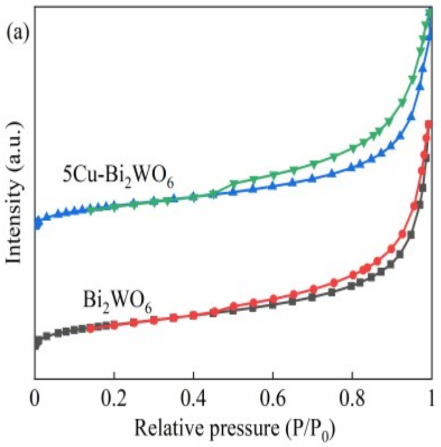

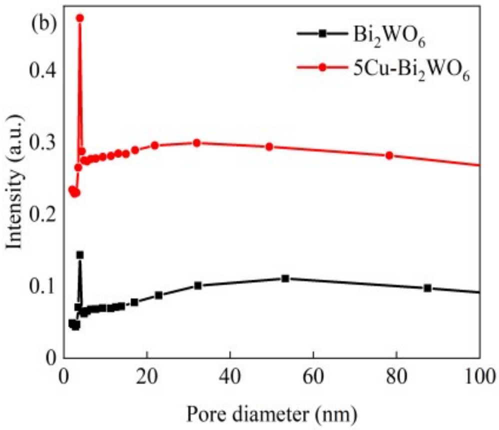

Figure S2 (a) N_2 adsorption–desorption isotherms and (b) pore size distribution of Bi_2WO_6 and 5Cu-Bi_2WO_6.

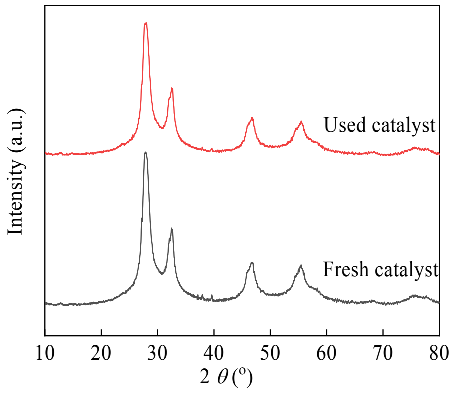

Figure S3 XRD spectra of 5Cu-Bi_2WO_6 before and after reaction.

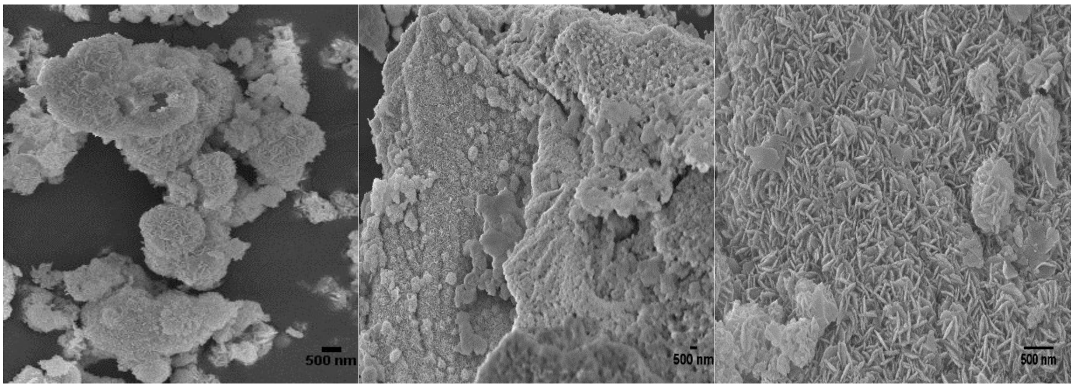

Figure S4 SEM images of 5Cu-Bi_2WO_6 after reaction.

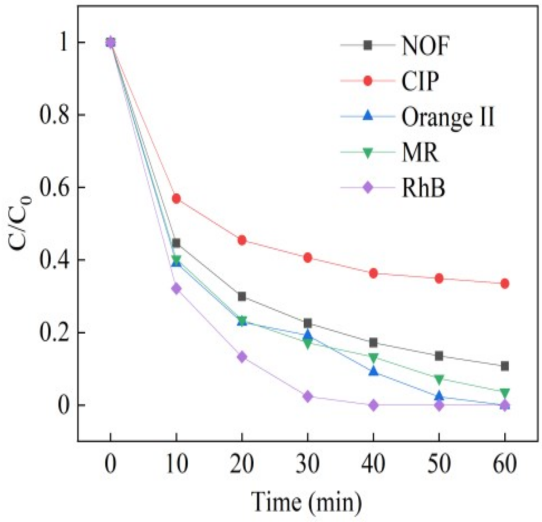

Figure S5 Degradation of different contaminant by 5Cu-Bi_2WO_6.

Table S1 Studies on of photocatalysts in the catalyst/PMS/PS/Vis reactions

No.	Systems	Light source	Target pollutant	Reaction time and efficiency	Operation parameters
1	PMS/$CuBi_2O_4$/VL	300W xenon lamp	TC 50 mg/L	Nearly complete removal 60 min	[PMS]= 0.125 mg/mL [catalyst]=0.5 g/L
2	PMS/Co-$BiVO_4$/VL	xenon lamp	Tetracycline hydrochloride 40 mg/L	Nearly complete removal 25 min	[PMS]= 5 mM [catalyst]=0.2 g/L
3	MIL-53(Fe)/PS/VL	300W xenon lamp	tetracycline hydrochloride 300 mg/L	99.6% TC 60 min	[PMS]= 8 mM [catalyst]=0.2 g/L
4	solar/MOFs@COFs/PS	500 W Xenon lamp	bisphenol A 50 mg/L	82% 120 min	[PMS]= 0.5g /L [catalyst]=0.25 g/L
5	Vis/TiO_2/PMS	300 W Xenon lamp	Perfluorooctanoic 50 mg/L	100% 9 h	[PMS]= 0.75 g L^{-1} [TiO_2] = 0.25 g L−1
6	Vis/Fe(II)/V(IV) self-doped $FeVO_4$/PMS	300 W Xenon lamp	Sulfamethoxazole 0.02 mM	96.6% 60 min	[catalyst] = 0.5 g L^{-1}, [PMS] = 0.406 mM
7	Vis/MoS_2/Ag/g-C_3N_4/PMS	300 W Xenon lamp	Tetracycline 20 mg/L	79.7% 50 min	[PMS]= 0.1 mM [catalyst]=0.2 g/L
8	Vis/ Fe/C_3N_4/PS	350 W Xenon lamp	rhodamine B 20 mg/L	100% 40 min	[catalyst] = 400 ppm, [PS] = 3 mM pH = 3.5
This work	Cu-Bi_2WO_6+Vis+PMS	30 W LED lamp	Norfloxacin 20 mg/L	89.27% 60 min	[catalyst]=0.5 g/L [PS]=0.4 mM Neutral pH

[1] J. Zhang, C. Zhai, W. Zhao, Y. Chen, R. Yin, L. Zeng, M. Zhu. Insight into combining visible-light photocatalysis with transformation of dual metal ions for enhancing peroxymonosulfate activation over dibismuth copper oxide. Chemical Engineering Journal, 2020, 397, 125310.

[2] Xin Chen, Jiabin Zhou, Tianlei Zhang, Lidan Din. Enhanced degradation of tetracycline hydrochloride using photocatalysis and sulfate radical-based oxidation processes by Co/$BiVO_4$ composites. Journal of Water Process Engineering, 2019, 32, 100918.

[3] Ying Zhang, Jiabin Zhou, Junhui Chen, Xiaoqiong Feng, Weiquan Cai. Rapid degradation of tetracycline hydrochloride by heterogeneous photocatalysis coupling persulfate oxidation with MIL-53(Fe) under visible light irradiation. Journal of Hazardous Materials, 2020, 392, 122315.

[4] Shi-WenLv, Jing-MinLiu, Chun-Yang Li, Ning Zhao, Zhi-Hao Wang, Shuo Wang. Two novel MOFs@COFs hybrid-based photocatalytic platforms coupling with sulfate radical-involved advanced oxidation processes for enhanced degradation of bisphenol A. Chemosphere, 2020, 243, 125378.

[5] Bentuo Xu, Mohammad Boshir Ahmed, John L. Zhou, Ali Altaee. Visible and UV photocatalysis of aqueous perfluorooctanoic

acid by TiO_2 and peroxymonosulfate: Process kinetics and mechanistic insights. Chemosphere, 2020, 243, 125366.

[6] Junlei Zhang, Wei Zhao, Zhi Li, Gang Lu, Mingshan Zhu. Visible-light-assisted peroxymonosulfate activation over Fe(II)/V(IV) self-doped $FeVO_4$ nanobelts with enhanced sulfamethoxazole degradation: Performance and mechanism. Chemical Engineering Journal, 2021, 403, 126384.

[7] Chongyue Jin, Jin Kang, Zhilin Li, Min Wang, Zengmin Wu, Yuanhua Xie. Enhanced visible light photocatalytic degradation of tetracycline by MoS2/Ag/g-C3N4 Z-scheme composites with peroxymonosulfate. Applied Surface Science, 514, 2020, 146076.

[8] Hamed Heidarpour, Mohsen Padervand, Mohammad Soltanieh, Manouchehr Vossoughi. Enhanced decolorization of rhodamine B solution through simultaneous photocatalysis and persulfate activation over Fe/C_3N_4 photocatalyst. Chemical Engineering Research and Design, 2020, 153, 709-720.

Table S2 Possible intermediates of NOF during the reaction

Name	m/z	Structure
NOF	m/z=320	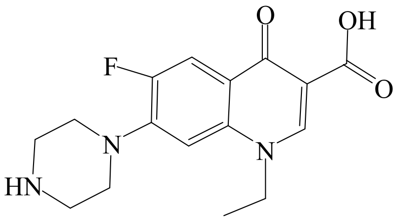
N1	m/z=350	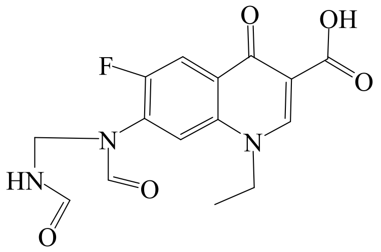
N2	m/z=336	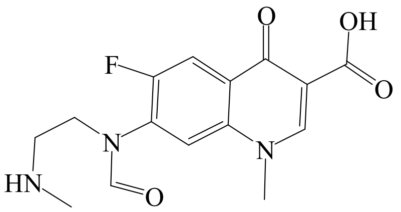
N3	m/z=322	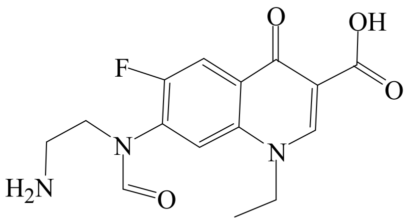

N4	m/z=294	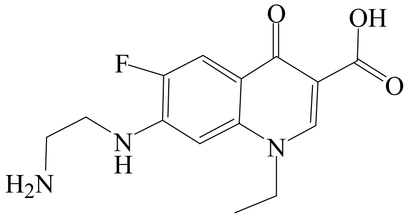
N5	m/z=279	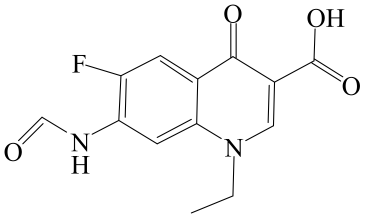
N6	m/z=251	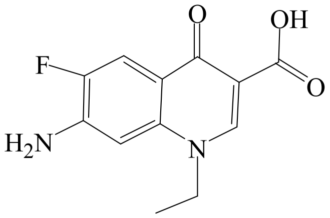
N7	m/z=233	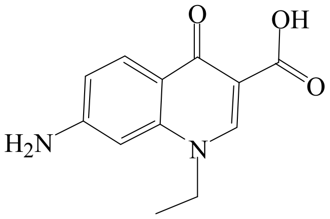
N8	m/z=304	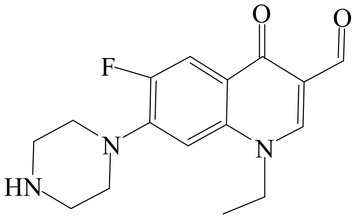
N9	m/z=276	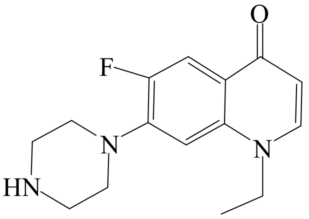
N10	m/z=302	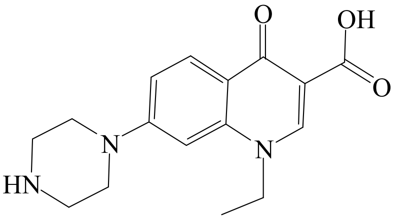

N11	m/z=318	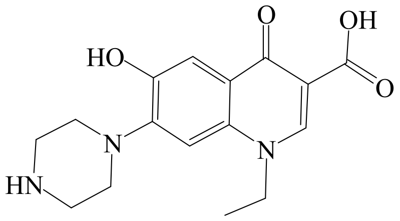
N12	m/z=274	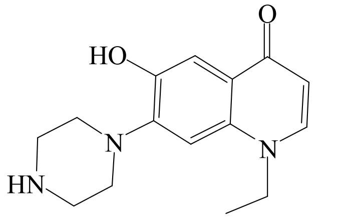
N13	m/z=352	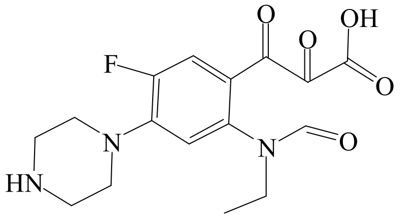
N14	m/z=302	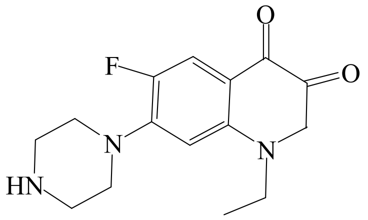
